# Recommendations on How to Use Flight Initiation Distance Data in Birds

**DOI:** 10.3390/biology14040329

**Published:** 2025-03-24

**Authors:** Magne Husby

**Affiliations:** Section of Science, Nord University, 8049 Bodø, Norway; magne.husby@nord.no

**Keywords:** alert distance, canoeing disturbance, dog disturbance, drone disturbance, FID, flock size, human disturbance, nature management, shorebirds, urban–rural, waterbirds

## Abstract

Birds and other wildlife are negatively affected by many anthropogenic activities, including human recreational activities, which are often not considered in area planning. Here, I present factors affecting the flight initiation distance (FID)—the distance to an approaching human at which birds flee—for 1075 different flocks of waterbirds. The FID varied greatly between groups of birds and species. For some bird groups and species, the FID was longer in rural areas than in urban areas and increased with flock size and with disturbance from canoeing. In addition to the differences in FID between species and groups of species, there are two important conclusions from this study: (1) a graphical relationship between the proportion of birds that flee at different distances from an approaching person gives more information than mean or median FID values and should be used by nature managers, and (2) the FID should be investigated in each area before mitigating actions or new constructions are decided, considering all the factors affecting it. A global database with a mixture of FID values from a huge number of areas is valuable for some purposes but can be misleading for individuals in a specific area.

## Simple Summary

Data on flight initiation distance (FID) is an important tool in bird conservation to reduce the disturbance caused by human recreational activities. FID vary substantially between species and areas, and should be investigated in all areas before mitigation action or new constructions are decided. Instead of using mean or median FID values, I recommend the construction of a more accurate graphical relationship between the proportion of birds that flee at different distances from an approaching person. This will give detailed and necessary information to nature managers.

## 1. Introduction

Birds are important components of ecosystems, providing essential ecosystem services [1], and their diversity is positively related to human mental health [2]. In addition, birds are monitors of environmental change that can inform us about changes in the natural environment [3,4]. As they are widely distributed, relatively easy to survey, and responsive to environmental changes, we know more about their taxonomic, functional, and phylogenetic diversity; geographic distributions; ecology; conservation status than for any other comparable group of organisms [5], and we often know why some bird populations vary in abundance and distribution. In some parts of the world, bird populations are extensively monitored to follow their trends, especially in Europe [6] and the USA [7]. The monitoring results show that many bird populations are declining. For example, there are 29% fewer breeding birds in the USA now compared to 1970 [8], and there has been about an 18% decline in the number of breeding birds in the European Union (EU) since 1980 [9]. Anthropogenic activity—either directly or indirectly—is the main explanation, and worldwide, one in eight bird species is threatened with extinction [10]. An important factor in conservation is the disturbance caused by human recreational activities, which is the focus of this publication.

Habitat destruction, fragmentation, and degradation have led to reduced bird populations through the loss of nesting sites, lower food availability, and increased predation risk [5,11,12,13]. Bird areas and their quality are reduced by, for example, urbanization and the expansion of roads, industrial areas, and buildings [14,15,16,17]; agricultural expansion and intensification [18,19,20,21,22,23]; drainage [24]; deforestation and forestry [12,25]. Other negative anthropogenic factors include pollution [12,26,27], climate change [28,29,30,31,32,33], wind farms and other kinds of energy production [34,35,36,37,38,39,40,41,42], collisions with buildings, powerlines, and cars [16,17,43,44,45,46,47,48,49], invasive alien species [11,12], and hunting and fishing [12,26]. The ultimate factors behind these and other destructive anthropogenic impacts on birds are the increases in human populations and the per capita rate of consumption [5].

The factors influencing bird populations mentioned above are not exhaustive. Another important factor is the disturbance caused by mere human presence in the environment. There is a substantial amount of disturbance from people in some important bird areas [50,51]. Birds should flee when approached by a possible predator; for example, a human that is viewed as a predator [52]. However, if a bird flees from an approaching animal that is not dangerous, this is a waste of time and energy. This might reduce the energy resources needed for other important activities and thus potentially reduce reproductive success and population size. Therefore, natural selection should result in an optimal flight initiation distance (FID)—the distance at which animals flee from an approaching danger—because of cost–benefit trade-offs. The FID will probably vary according to the kind of danger that is approaching and individuals’ earlier experiences at different locations. This variation minimizes the costs of disturbance and maximizes the chances of survival. The FID is, therefore, naturally influenced by human presence and many other different factors [53,54,55,56,57,58,59,60,61]. For example, many waterbirds are negatively affected by human presence as they are scared away from otherwise important feeding, roosting, or breeding areas. In addition, human presence changes birds’ activity patterns in a negative way [50,51,61,62,63,64]. In the last 50 years, shorebirds (44 species) in the USA have declined by 37.4% [8].

The FID is an important trait because escape behavior is vital for the survival of many species, and in the practical management of wild birds, knowledge about the vulnerability of different species to disturbances is vital. As birds’ responses to human disturbance are highly variable and may depend on many factors, FID values in one region might not necessarily be transferable to other regions. As a novel application of the FID, I use the relationship between the proportion of fleeing birds and birds that stayed at different distances from pedestrians to improve the evaluation of the negative effects of human presence. In addition, I investigated different factors affecting the FID in waterbird groups (swans, geese and shelducks, dabbling ducks, diving ducks, waders, and gulls) and at the species level (Eurasian teals *Anas crecca*, mallard *Anas platyrhynchos*, common eiders *Somateria mollissima*, common goldeneye *Buchephala clangula*, and Eurasian oystercatcher *Haematopus ostralegus*) depending on data availability. Due to individual variations in the FID between species, determining the FID at the species level is important for conservationists.

## 2. Materials and Methods

### 2.1. Field Methods

**Figure 1:**
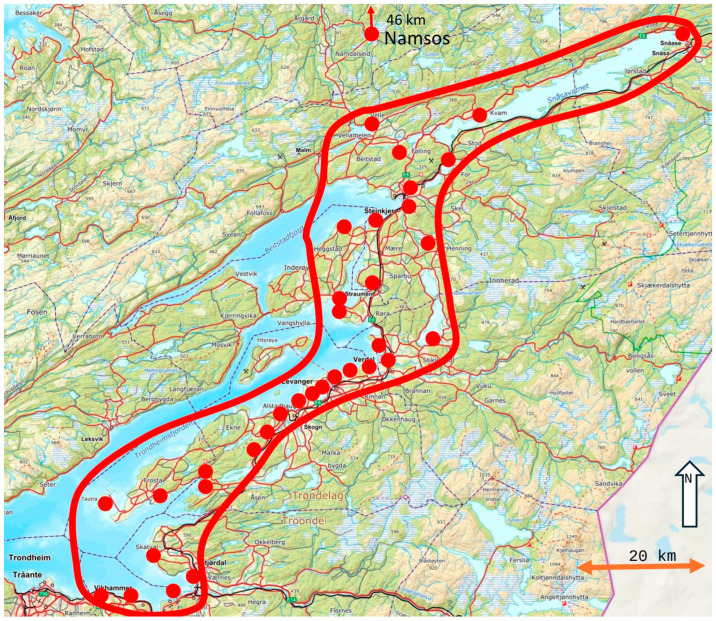
The study area in the middle part of Norway (north of Trondheim), showing each of the study sites, except one site situated further north, as illustrated by the arrow.

The study area was in the middle part of Norway (Figure 1) and included 35 freshwater and saltwater areas of various sizes and distances from urban areas that were investigated between 2001 and 2019. All urban areas in this study were outside the centers of small cities and should be considered as suburban areas. Many areas were included to reduce the probability that the same birds could be studied several times. One or two researchers walked directly toward a focal bird flock (1–700 individuals) to avoid potential variations in the FID caused by a change in direction and because a tangential approach might reduce the FID compared to walking directly toward the birds [59,65,66]. I also started my approach far away from the birds in an open landscape when they were engaged in normal activities—most often searching for food—to avoid possible problems related to the positive relationship between the researcher’s starting distance and FID [67,68]. This was performed in an open landscape to ensure that the birds detected us at a long distance and that no birds had a short FID because they were startled by us [69]. The sailboat, canoe, and drone were directed directly against the birds.

Altogether, the FID was measured for 1075 different flocks of waterbirds: swans and geese (*n* = 87), dabbling ducks (*n* = 323), diving ducks (*n* = 386), mergansers (*n* = 42), waders (*n* = 137), and gulls (*n* = 100). Included in the analyses are anthropogenic disturbances from humans without a dog (*n* = 767 bird flocks), humans with a leashed dog (*n* = 140), the use of a canoe and sailboat (*n* = 133), and the use of a drone (*n* = 35). A flock is defined as one or more birds of the same species separated by about 50 m or more from other birds of the same species. The exact number of flocks in each size category is given in Table 1. Most birds were approached while they were searching for food because loafing birds may have different FIDs to birds searching for food both within and between species [70]. The FID was measured by the researchers (one or two) walking slowly (about 0.7 km/h) and directly against the flock of birds. This standardization is necessary as fast movements increase the probability that the birds will flee [51]. The researchers always observed the birds to follow their behavior; however, the FID might increase if we looked intently at the birds compared to looking in another direction [71].

The researchers stopped and measured the distance to the focal bird flock when a change in behavior was registered—for example, when the birds stopped feeding or became alert or moved away—to roughly measure the distance. If the flock consisted of more than one bird, special attention was paid to the closest bird, and distance measurements were taken with reference to this bird. This bird also generally seemed to change behavior first. The slow walking and stopping behavior of the researchers is similar to that of birdwatchers. Some birds (4 of 20 species) have been found to have a longer FID as a response to this behavior compared to their FID to people who are only walking without stopping and looking in binoculars [72]. Early in the investigation period, some alert distances were noted; that is, the distance from the researcher when the birds stopped their normal activity. When the birds fled, the researchers stopped walking and, for about half of the registrations, noted how far away the birds moved from their starting point. The researchers retreated, and, in a few cases (*n* = 90), they observed exactly when the fleeing birds moved away and when they came back to the place they started from. However, many flocks did not return. All distance measurements were made with laser instruments (Swarovski EL Range 8 × 42, Austria or Nikon Laser Rangefinder Prostaff 5, Tokyo, Japan).

The fieldwork was performed throughout the day in good light conditions in winter and early spring (January *n* = 36 flocks, February *n* = 114, March *n* = 87, April *n* = 195, May *n* = 73, and December *n* = 63), and autumn (August *n* = 78, September *n* = 200, October *n* = 118, and November *n* = 111). No strongly colored clothing was worn; it was normally green field clothing.

The drone (DJI Phantom 2 Vision +) was operated with a speed of 2 m/s and an approximately 10 m flight height above the birds situated on the ground or on the water, away from the other experiments. FID measurements using a canoe and sailboat were only performed in one area, and these measurements were analyzed separately and not included in the generalized linear mixed model (GLMM) analyses. This area was open, quite rectangular, and about 1 × 2 km wide, meaning the birds should have been able to detect the boat long before they decided to flee. All measurements were performed in good weather conditions with little wind and no or very little precipitation. No breeding birds were included in the experiments.

The fleeing method was categorized as flying away or walking/swimming away. Walking and swimming were combined because both running and swimming birds with a similar mass use a comparable amount of energy at maximum speed, while the energy consumption during flight is approximately 2.5 times greater. This difference is caused by the higher mass of the flight muscles compared to the leg muscles, as well as their greater oxidative capacity [73].

### 2.2. Statistics

Due to non-normal data, the differences in FID between groups of birds or between species were tested using the non-parametric Mann–Whitney U-test (MW U-test). Likewise, the relationships between FID values were tested with non-parametric Spearman rank correlation tests (r_s_). The variables included in the GLMM analyses are given in Table 1. The maximum correlation between the six explanatory variables (Table 1) was 0.343, far below the recommended upper limit of 0.7 [74].

Generalized linear mixed models (GLMMs) were used [75] because they remove the variability in responses that is associated with random factors other than the conditions of experimental interest, thus reducing the Type I error rate [76]. GLMMs may be the best tool for analyzing non-normal data that contain random effects [77]; however, the FID values were normalized using log_10_ in our GLMM analyses. From the global model with log_10_FID as a target variable [78], I selected various candidate models with the explanatory variables included without interactions by using backward stepwise removal of explanatory variables and Akaike’s Information Criterion (AIC). The best models had a difference in the AIC values (△AIC) below the upper recommended value of 2, while models with △AICs > 2 compared to the optimal model were excluded [78,79]. In the GLMM analyses with log_10_FID as the target variable, I used a normal distribution and the identity link function. With the fleeing method as the target variable, I used a binary probability distribution with the logit link function and added log_10_FID as an explanatory variable. The period was included in the GLMM analyses as a random factor because all registrations were outside the breeding and main molting seasons, and potential differences in FID during autumn, winter, and early spring are of minor interest in this investigation. However, this potential variation should be compensated for. An attempt to include site as a random factor was not accepted by SPSS (ver. 30), probably because some sites had very few FID observations.

Due to the low number of measurements for each species, the effect of the drone on the FID was analyzed separately and not included in the GLMM analyses. The drone experiments were performed in a few places with a minimum of 5 km between each area, and these areas were not used in the FID experiments with pedestrians.

Using graphical tools [79], the histogram with Regression Standardized Residuals and Frequency showed a normal distribution [76] for the log_10_FID values. Data exploration [77,78,79] revealed no outliers. The effects of the use of a canoe and sailboat on FID were combined because there was no significant difference between them (MW U-test: z = −0.738; *n* = 117 and 16, respectively; *p* = 0.460), and they were analyzed separately from the GLMM because they were present in only 1 of the 35 areas. Likewise, the effects of an approaching drone (*n* = 35) were analyzed separately due to the lack of data and the low number of tested areas.

Statistical tests were two-tailed with an *α*-level of 0.05 (*p* < 0.05). I am aware of the ongoing discussion about null hypothesis and statistical testing (NHST) versus IT-based inference [80], but I found the data to be suitable for NHST methods [78]. SPSS was used for all statistical calculations.

## 3. Results

### 3.1. FID Variation Between Different Waterbirds

**Figure 2:**
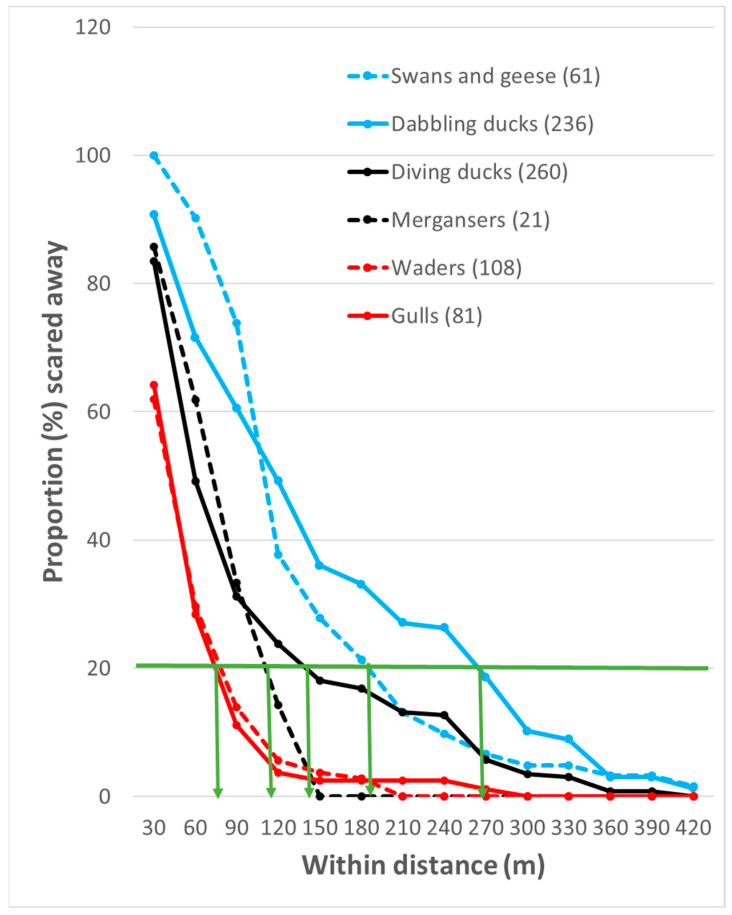
Proportion (%) of bird flocks belonging to different bird groups that fled when approached by pedestrians on land (without a leashed dog) at various distances. The number of flocks of each group is given in brackets. The green line indicates the point at which 20% of the birds fled, and the green arrows show the distance from the human at which this occurred for different bird groups.

The six different groups of birds exhibited clear differences in flight initiation distance (FID) when the researchers approached via walking on land without a leashed dog (Figure 2). Waders and gulls had similar FIDs, shorter than the other groups. The FID (log_10_FID) was significantly shorter for waders than for mergansers (MW U-test: Z = −3.221; *n* = 108 and 21, respectively; *p* = 0.001) and for gulls than for mergansers (Z = −3.300, *n* = 81 and 21, *p* < 0.001). Mergansers and diving ducks had similar FIDs (Z = −0.642, *n* = 21 and 260, *p* = 0.521). Dabbling ducks had a significantly longer FID than diving ducks (Z = −6.250, *n* = 236 and 260, *p* < 0.001). Swans/geese and dabbling ducks had similar FIDs (Z = −0.068, *n* = 61 and 236, *p* = 0.946).

Figure 2 shows the proportions of birds that were scared away when humans were at different distances from them. It is possible to use this figure to observe the effects of human presence in more detail than when using mean and median values only. As an example, a horizontal line is shown in the figure to indicate when 20% of the birds were scared away, with the arrows from the interception of this line and the lines for different groups of birds indicating at what distance this occurred. In the example, 20% of the waders and gulls were scared away when humans were present at around 75 m away; mergansers and diving ducks around 120–140 m, and swans and geese, and dabbling ducks around 190–270 m away. If the accepted proportion of birds scared away is lower than 20%, the distances to humans are greater.

**Figure 3:**
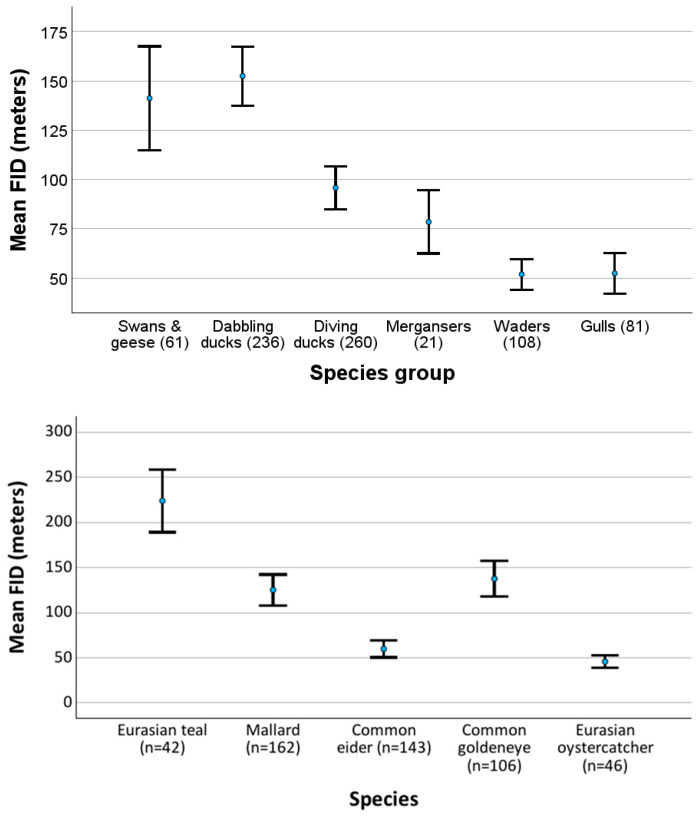
Flight initiation distance (FID) (±2 SE) for six different groups of waterbirds (**upper**) and five species (**lower**). The number of flocks is given in brackets. All birds were approached by a pedestrian on land, without a dog.

The mean FIDs for different groups of birds and some of the species are given in Figure 3 when the disturbance was a pedestrian on land without a dog. The mean FID was about 150 m for swans, geese, and dabbling ducks, less than 100 m for diving ducks and mergansers, and just above 50 m for waders and gulls (Figure 3). At the species level, there were also quite large differences (Figure 3). Eurasian teals were shy and moved away when humans were about 225 m away, while mallards and common goldeneyes had a mean FID of about 130 m, and common eiders and Eurasian oystercatchers had a mean FID of about 50 m. If this mean value of 50 m is taken to create a buffer against human presence for waders, nearly 40% of them would be scared away from the area (Figure 2).

### 3.2. Factors Affecting the FID and Fleeing Method

The next best model in the GLMM analyses with log_10_FID as a target variable, with an △AIC = 1.0 compared to the optimal model, included all explanatory variables in Table 1, while the optimal model excluded the variable narrow or wide area. These two models were the only relevant models. The model containing all explanatory variables was used. Table 2 and Table 3 show the results of the GLMM analyses of the factors included in this model affecting the FID for both groups of birds and at the species level, respectively. Due to the low number of observations (*n* = 26), I did not run the GLMM analysis for mergansers. Two researchers approaching together (compared to only one researcher) led to a shorter FID for dabbling ducks when all other variables in the analysis were taken into consideration (Table 2). This effect was not significant for any of the other four groups. For species with at least 50 observations, two researchers approaching them resulted in a shorter FID for Eurasian teals and mallards but not for common eiders, common goldeneyes, or Eurasian oystercatchers (Table 3).

Narrow water areas led to a shorter FID compared with wide areas for both dabbling ducks (including mallards) and diving ducks (including common eider), but the opposite was true for Eurasian oystercatchers. For diving ducks, including the common goldeneye and gulls, for some flock sizes greater than one individual, there was a longer FID compared with one individual, while the FID was shorter for flocks of two individuals compared with one individual for Eurasian oystercatchers. When the researchers were not accompanied by a dog, both dabbling and diving ducks, including common goldeneyes, had a longer FID compared to when the researchers were accompanied by a leashed dog, and again, the opposite was true for Eurasian oystercatchers. In suburban areas, a shorter FID was found only for diving ducks, including the common eider, compared to rural areas.

**Figure 4:**
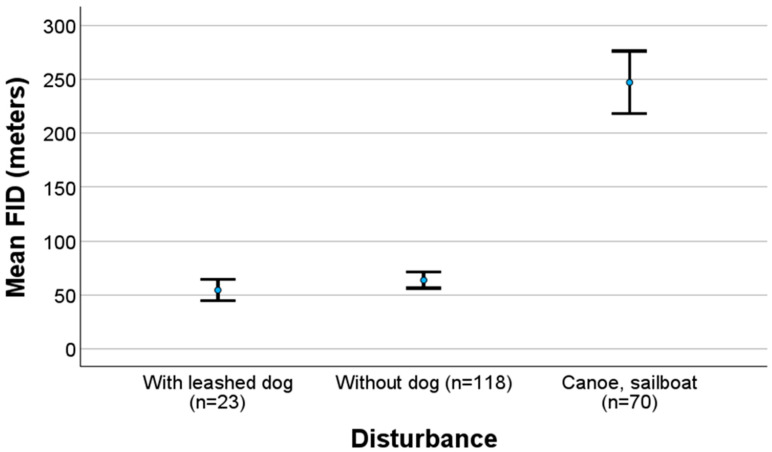
FID (±2 SE) for diving ducks with two kinds of disturbances from a pedestrian and from the use of a canoe/sailboat in the area where the effects of canoeing and the sailboat were investigated.

The FID test with canoe and sailboat was only performed in one area, and the results are presented using diving ducks as an example in Figure 4. There was no significant difference in FID when the researcher was accompanied by a leashed dog compared with the researcher without a dog (MW U: z = −1.178, *n* = 23 and 118, *p* = 0.239) in this area. The use of a canoe and sailboat significantly increased the FID both for the researcher accompanied by a leashed dog (MW U: z = −6.560, *n* = 70 and 23, *p* < 0.001) and the researcher without a dog (MW U: z = −10.099, *n* = 70 and 118, *p* < 0.001). Canoeing and the use of a sailboat led to similar FIDs (see Statistics). The finding of a similar FID when the researcher was or was not accompanied by a leashed dog is opposite to those when I used the whole dataset (Table 3); however, in the GLMM analyses (Table 3), it was accounted for the other variables in the models.

The FID was shorter when a drone approached whooper swans *Cygnus* cygnus, pink-footed geese *Anser brachyrhynchus*, graylag geese *Anser anser*, common shelducks *Tadorna tadorna*, mallards, tufted ducks *Aythya fuligula*, common eiders, common goldeneyes, and red-breasted mergansers *Mergus serrator* compared with the FID when one or more people approached the same species by walking towards them without a dog. It was not possible to compare the mean values of log_10_FID for all species due to the lack of data, but the difference was near-significant for pink-footed geese (*p* = 0.054) and significant for mallards (*p* = 0.038) and common goldeneyes (*p* = 0.021). Despite the lack of statistical tests, it is apparent that in general, swans, geese, and ducks are less afraid of an approaching drone than an approaching pedestrian. Eurasian oystercatchers had a near-identical FID when the two disturbances were compared, and the common gull *Larus canus* tended (*p* < 0.1) to have a shorter FID when a drone was approaching compared to a pedestrian. In the tests, equal variances were not assumed.

In the GLMM analysis with the fleeing method as the target variable, the best models excluded the explanatory variables of open/narrow water (△AIC = 4.7 from the optimal model) and flock size (△AIC = 2.9). The next best model, with an △AIC = 1.5 from the optimal model, excluded rural/urban areas, while the optimal model excluded disturbance. Two relevant models had △AICs < 2 from the optimal model. I ran the analyses including all variables in the next best model (Table 1, only excluding open/narrow and flock size), and found that relatively more birds flew away compared to swimming or walking away in rural areas compared to urban areas (*p* = 0.036). All the other variables included in the final model, including log_10_FID (*p* = 0.328), had no significant effects on the fleeing method.

The fleeing method was analyzed for all birds belonging to the six bird groups; the disturbance was a researcher without a dog (*n* = 500). There were clear differences in the proportion of the individuals that flew away between the different groups of birds or species compared with the proportion that swam or walked away. In the smaller wader and gull species, 82% and 81%, respectively, flew away, while 67% of the dabbling ducks and only 26% of the diving ducks flew away. Mergansers exhibited similar behavior to dabbling ducks (73%, but there were fewer observations), and 46% of swans and geese flew away. The proportion of individual species that flew away was similar to that of the bird groups they belonged to (data not presented).

For swans/geese, dabbling ducks, and diving ducks, the FID was significantly longer in birds that flew away compared to birds that swam or walked away from the human disturbance (Table 4). In the other bird groups, there was no such tendency, but the number of measurements for mergansers was too low to draw a meaningful conclusion.

### 3.3. The Relationship Between FID and Other Behaviors

Before the birds started to move away from the researcher, they showed alert responses, which most often meant that they stopped feeding. There was a significant positive correlation between the alert distance from the researcher and the FID in all tests with sufficient data (Table 5). It is also interesting to note that dabbling ducks, diving ducks, mallards, and common eiders with a long FID moved significantly further away from their starting point than birds with a shorter FID (Table 5). That means that shy birds with long FIDs lose more feeding time and use more energy than more confident birds.

There was no significant correlation between FID and how many minutes it took before the birds came back to where they fled from for all measurements combined (*n* = 90), but the number of data is low. Near-significant correlations (*p* < 0.1) were found for dabbling ducks, mallards, and common eiders, but the directions of the relationships were not consistent (Table 5). Birds that did not return during the registration period, either because they started to search for food in another area or because they went out of sight, were not included.

## 4. Discussion

### 4.1. FID Variation Between Different Waterbirds

I found the longest FID for swans/geese and dabbling ducks, while the FID was shorter for diving ducks and mergansers and the shortest for waders and gulls. This is in accordance with other findings that vegetarian birds that consume immobile food have a longer FID than birds that consume mobile food, which can be difficult to catch [70]. My findings are also mainly in accordance with earlier investigations demonstrating that larger birds have a longer FID than smaller birds [81,82,83,84,85,86]. However, within dabbling ducks and diving ducks, the smallest species had the longest FID, which is in accordance with one investigation that showed that large birds are more tolerant than small birds [87]. The authors suggested that the reason for their result is that larger bird species might be under intense pressure to be more tolerant of humans due to the high energetic costs of unnecessary escape. In addition, it is proposed that larger birds may be less likely to be killed by predators because of their larger body size and their relatively larger brains with potentially greater cognitive abilities that might be more able to assess risks [88]. However, these possible explanations should also be valid in most other areas. In the areas investigated in this study, there are many hunters that hunt geese [89] and ducks, and there is a dense population of white-tailed eagles *Haliaeetus albicilla* that also hunt the largest birds. The high energetic expenditure during flight for waders [90] might explain why the FID was shorter for this group of birds than most other groups (Figure 2 and Figure 3).

I did not use any statistical tool, for example, Moran’s I, to demonstrate possible spatial autocorrelation. There was a lot of variation in FID between areas, and the FID in one area did not seem to affect the FID in other areas. Even neighboring marine areas differ substantially because of variations in salinity, exposure to wind and waves, and substrate, and therefore also species composition and FID values.

### 4.2. Factors Affecting the FID and Fleeing Method

For dabbling ducks (both Eurasian teals and mallards), when only one researcher approached, the FID was longer than for two researchers (Table 2 and Table 3). This is opposite to what was expected. The reason for this might be because two or more people walking together is very common in the investigated areas, and one person alone is typical behavior of hunters of these species.

Shy birds have a long FID, and it is logical to expect that they will fly away from a human disturbance more often than walking or swimming away. For all birds combined, there was no such relationship, probably because the shyest bird groups consisted of heavy birds with high flying costs, and they could take advantage of their long FID by swimming or walking away to save energy. Most smaller birds, such as gulls and waders, saved energy by allowing an approaching human to come close, but they had to fly away when the threat felt too dangerous. Within each of the three most shy bird groups (Figure 2), where the birds are more similar in size, there is a significant positive relationship between the FID and the proportion of the birds that flew away (Table 4). To conclude, saving energy seems crucial when a bird decides to fly or swim/walk away from an approaching pedestrian. A long FID makes it possible to escape by less costly swimming or walking (swans/geese, dabbling and diving ducks); alternatively, a short FID may mean that the birds do not have to flee at all, but they must escape by costly flying if fleeing becomes necessary (gulls, waders). These two alternative strategies are probably affected by other factors, such as a combination of preferred food [70] and a relatively large body mass, making the proportion of diving ducks that flew away very low compared with other groups. Further research is recommended on this topic.

In narrow areas with <50 m to the opposite seashore, nearly all significant results showed that the FID was shorter here than in more open areas. This was true for dabbling ducks (including mallards), diving ducks (including common eiders), and Eurasian oystercatchers. This is probably because these narrow areas are not used by shy birds that normally would use a lot of energy by flying away more often relative to more confident birds. The FID of gulls was increased in narrow areas, probably because their short FID enables relatively shy individuals to stay in narrow areas. As expected, others have found that birds closer to the path were more likely to fly away as people approached than birds that were further away [91]. My findings of a short FID in narrow areas are opposite to those of an investigation in which the FID was investigated by use of a motorboat in lakes and rivers [68].

In the literature, the relationship between flock size and FID remains unclear [92]. Many studies have demonstrated that the FID increases with an increase in flock size [57,68,83,84,93,94]. This is not always the case, as flock size was found to have little effect on FID in a review paper featuring 99 bird species, but there was a positive relationship in waders [92]. However, other investigations have found that FID increases with flock size in only one of nine waders [95] and in two of five waders [96]. In the present publication, there were not many significant relationships between flock size and FID (Table 2 and Table 3). Only diving ducks, including common goldeneyes, and gulls had longer FIDs for some flock sizes that were larger than one individual. Eurasian oystercatchers had a shorter FID when there were two individuals in the flock compared to one in this study and considering the contrasting results of other investigations, further research is recommended for waders. There may be several explanations for the lack of generality in the finding of the effect of flock size on FID. For example, larger flocks have more individuals that can detect a predator, and therefore it is reasonable to expect that the FID will increase with flock size. There will be an opposite effect on the FID from dilution or safety in numbers, meaning that the probability that a certain bird is taken by a predator gradually reduces with flock size compared to a single bird, and thus, the FID might be reduced.

Dabbling and diving ducks, including common goldeneyes, had an increased FID when a pedestrian was not accompanied by a dog compared with when they were accompanied by a leashed dog (Table 2 and Table 3). For some of the investigated waders, a dog on a leash increased the FID [95], as observed for Eurasian oystercatchers in this investigation (Table 3). However, free-ranging dogs are found to scare birds more than leashed dogs [97]. In one publication, free dogs scared birds in a similar manner to construction vehicles and military jets, and more than gunfire and passing cars [98]. The waders did not return to their feeding ground after five minutes when disturbed by humans, but gulls did, and waders responded especially strongly if disturbed by a dog [63]. Western snowy plovers *Anarhynchus nivosus*, on their wintering grounds, are more likely to fly away from dogs, horses, and crows than from humans and other shorebirds [99].

In the suburban areas included in this study, I found that the FID was shorter only for diving ducks, including common eiders. In addition, other investigations show that birds adapted to human presence in urban areas are more tolerant (shorter FID) than birds in rural areas [55,83,87,100,101,102,103,104,105,106,107]. Despite this habituation, birds tend to overestimate rather than underestimate the risk associated with human presence [61]. Except for city parks in which some people feed the birds, birds will also fear people close to them and eventually flee. Variation in the number of people using trails was not found to influence the number of waders [108]. This might be because the FID for waders is relatively short, and peoples’ movement patterns when following tracks are predictable. However, the number of plovers was found to be lower near trailheads than elsewhere [99]. I expected to find a stronger urban/rural effect on FID in this investigation. This might be because the cities in this study are small and because the investigated urban areas are outside the densest part of the city in the suburban environment; and thus, the number of pedestrians here is not very high (but higher than in the investigated rural areas). Within cities, birds have a shorter FID in areas with a high pedestrian density [101], and urbanized birds have shorter FIDs than suburban birds [87].

Little is known about FID and recreational canoeing [109]. My investigation in an open area showed that the FID increased with the use of canoes and sailboats compared with pedestrians (Figure 4), in contrast to an investigation with paddling in a river [109]. When compared with pedestrians, a longer FID with the use of a boat was also found in pied avocet *Recurvirostra avosetta*, while a shorter FID was found in Eurasian oystercatchers [96]. More investigations are needed to compare the FID in the presence of canoes, paddling, and sailboats with that for pedestrians.

There are many investigations about the effect of an approaching drone on waterbirds [110]. These investigations have shown mostly shorter FIDs, similar to the results of this study, and drones are recommended for many waterbird censuses [111,112,113]. However, a considerable disturbance in waterfowl has also been observed, and it is recommended that drones should be forbidden in certain areas to minimize this potential disturbance [114].

### 4.3. Effects of Human Disturbance on Population Sizes

When birds are scared away from important feeding areas, the reduced food intake and extra energy expenditure can reduce their condition, survival, and population size. European birds with a long FID have more negative population trends than birds with a short FID [115]. When waders are forced to make 10 flights from their roosts per day, their daily energy expenditure increases by 4.5% and 7.8% (knots *Calidris* spp. and sand plover *Charadrius* spp., respectively), which may reduce energy reserves to levels below the thresholds that can be replenished by normal intake rates and thus negatively affect survival [116]. Another investigation showed that the effects of disturbance are dependent on the number of pedestrians, and very few pedestrians per day do not seem to have any effect on waders’ energy budget [82], with the authors calculating that the number of disturbances needs to be about 100–300 per day for different wader species before the feeding time is reduced by 10%, thus contrasting the investigation on knots and plovers referred to above.

Waders inhabiting tidal areas do not feed at full tide, and during the winter, waders the size of redshank *Tringa totanus* and smaller use 90–100% of daylight time, while larger waders use 50–80% of daylight to search for food when feeding grounds are exposed [117]. It is, therefore, reasonable to believe that disturbances that reduce feeding time and increase energy expenditure have a negative effect on birds. Piping Plovers *Charadrius melodus* on a beach with few people devote more time to foraging and less time to vigilance than birds at a beach with more people [62], which was also observed for waterbirds [91]. Sanderlings *Calidris alba*, feeding on the beach and running or flying away from people up to five times per minute, were searching for food during the evening and night when human disturbance was low [93]. Individually marked oystercatchers do not increase their feeding rate to compensate for lost feeding time; instead, they extend their feeding time by remaining longer on the mussel bed. These oystercatchers were able to compensate for short time losses of 30–60 min through disturbance [118].

To evaluate the negative effects of human disturbance, it is necessary to take into consideration the alert time, feeding time, and fleeing method, as well as the fact that the other area might have less food than the area they flee from. This investigation shows a positive correlation between FID and alert distance, fleeing distance, and a longer wait before they came back to their feeding grounds (Table 5). A positive relationship between FID and fleeing distance was also found by others [119]. This implies that shy birds with a long FID lose a lot of time and energy. However, extra loads in a period might be camouflaged by higher feeding rates or longer feeding periods, as referred to above, and therefore, individually marked birds must be investigated for a long time to unmask the negative effects of disturbance on population sizes. Experiments have shown that parent birds that were given extra loads managed to raise their nestlings to fledge but were less able to raise their fledglings to independence than control parents [120]. Such mechanisms make it challenging to quantify the impact of human disturbance in stopover and wintering sites on shorebird population sizes. So far, surprisingly little has been uncovered in this field [121].

### 4.4. Could a Worldwide FID Database Be Useful?

From the literature, it is possible to obtain FID information from a huge number of bird species around the world. In fact, a review paper published a database of 34,775 FIDs from 50 studies representing 650 non-nesting bird species from 2009 to 2015 [122]. The FID values in that publication were, in general, much lower than in this investigation, probably because investigations in all kinds of areas were included in the worldwide list, for example, parks in cities with many more people than in my investigated area, and thus it includes individuals habituated to human presence. However, large-scale FID databases can provide valuable general trends, such as the database containing 10,249 observations for 842 bird species inhabiting open tropical ecosystems in Africa, South America, and Australia [123] and that summarizing the analysis of latitudinal variation in FID using observations of 12,495 FIDs of 714 populations of 159 species [55].

The FID is influenced by a lot of different factors, as shown in this and other publications. In addition, the FID has been found to be positively correlated with the abundance of predators [55], the number of blood parasites [56], genetic variation [124], and relative eye size [125], for example. The great variability in FID, caused by different factors that might vary geographically [55], suggests that wildlife managers should be somewhat conservative in developing buffer zones [53]. In my opinion, it is not appropriate to use previously published FID data for a given species as guidelines for setting buffer zones in a certain area. Each area should be investigated to determine the FID for different species at that actual site and the proportion of each species that is scared away at different distances by disturbances and to decide what level of disturbance is acceptable (Figure 2). If the area is used by rare or red-listed species, the acceptable level of birds scared away should be zero or close to zero. FID investigations should be conducted even though shy bird species with a long FID are consistently shy also in other areas, while more tolerant species are consistently tolerant [53]. Not all species are investigated with regard to their FID, leading to a taxonomic bias in available FID values, and the available values are not often used by decision-makers [86]. This conclusion supports my recommendation that new site-specific investigations should be carried out in each actual area, with the relevant type of disturbance.

### 4.5. Conclusions

Graphs showing the relationship between different birds’ probability to flee in relation to the distance from pedestrians (Figure 2) give more detailed information about bird behavior than median or mean FIDs. I, therefore, recommend the construction of similar graphs for each area to evaluate mitigation actions or whether a trail should be constructed or not. As far as I know, such graphs have not yet been constructed for this purpose; however, they have been constructed for a few bird species to evaluate the effects of pedestrians alone compared with pedestrians with dogs [97].

Nature managers and politicians should consider the negative effects of human presence on seashores and in and along lakes and rivers. On seashores, human recreational activities, such as fishing, walking, jogging, swimming, horseback riding, vehicle driving, dog walking (on and off leash), and kiting, occur mostly in the same zone of the beach used by waders, gulls, and terns [126]. In areas accessed by people, the closure of important wintering bird areas for humans has resulted in increased breeding populations [121,127], and appropriately constructed bird hides have reduced the disturbance for birds [128]. In areas where new activities are planned—for example, new trails along important areas for waterbirds—the species, their abundance, their FID, and the area’s ecological functions for the different species should be investigated. I recommend that (1) in each actual area, the FID is investigated by following the methods described in this and other publications; (2) a diagram is constructed to show the relationship between the distance from the birds and the proportion of birds of each species that flee (Figure 2 and Figure 3) a buffer zone is established based on the results during the time of the year when the birds are most vulnerable, as well as on their red list status. Thereafter, it should be determined whether the trail or other physical developments should be constructed or not.

In the literature, human disturbance is listed as an important conservation issue confronting shorebirds [121]. To reduce the conflict between human recreational activities and important bird feeding, roosting, and nesting areas, a map of all actual areas is necessary [129]. The species that are using the area and how the area is used (breeding, feeding, loafing, roosting) should be investigated. Wildlife managers should use the FID more than they do today to develop buffer zones to reduce human impacts on wildlife [130]. However, the FID varies between species and between sites [53], as found in this and other investigations. The great variability in FID implies that wildlife managers should be somewhat conservative in developing buffer zones. They can use previously published FID data for a given species as guidelines for setting buffer zones [53,85], but only if new investigations are not possible. Despite the challenging task of demonstrating the negative effects of human recreational activities near wetlands on bird conditions, survival, and populations, researchers should prioritize obtaining such important knowledge. Perhaps, in the future, fewer tracks will be constructed near the shorelines, wetlands, and rivers, without any concerns about the bird’s well-being.

## Figures and Tables

**Table 1:** Variables included in the generalized linear mixed models (GLMM) analyses. N is the number of flocks with FID as the target variable.

Variable	Value	Comments	N
**Target Variable**			
FID	Continuous	Log_10_FID	907
Fleeing method	1–2	1 = swimming or walking, 2 = flying	227, 353
**Explanatory variable**			
N of researchers	1–2	1 = one person, 2 = two people	720, 187
Open-narrow water	1–2	1 = open area, 2 = narrow (<50 m to the opposite shore)	664, 243
Flock size	1–7	1–4 = exact number of birds, 5 = 5–10 birds, 6 = 11–30, 7 = 31–700	327, 174, 78, 49, 126, 86, 57
Disturbance by dog	1–2	1 = with leashed dog, 2 = without dog,	140, 767
Rural–urban	1–2	1 = rural, 2 = urban	368, 539
**Random effect**			
Period	1–2	Winter and early spring, autumn	568, 507

**Table 2:** *p* values in the GLMM analyses with explanatory factors possibly affecting log_10_FID in different groups of waterbirds. If significant, the direction of the change is given by + or −. The effects of the explanatory variables (value written in brackets) are compared with the lowest value (=1) (see Table 1). For flock size, six different categories were compared with one individual and the lowest *p*-value is given. Significant results for the explanatory variables are in bold and * = *p* < 0.05, ** = *p* < 0.01, and *** = *p* < 0.001 for explanatory variables.

Variable	Swans and Geese	Dabbling Ducks	Diving Ducks	Waders	Gulls
Corrected model	0.898	**+<0.001**	**+<0.001**	0.587	0.144
Intercept	**<0.001**	**<0.001**	**<0.001**	**<0.001**	**<0.001**
N of researchers (=2)	0.795	−<0.001 ***	0.395	0.817	0.055
Open/narrow (=2)	0.257	−0.014 *	−<0.001 ***	0.510	+0.014 *
Flock size (2–7)	_Min_0.693	_Min_0.068	_Min_**+0.002** ^(a)^**	_Min_0.165	_Min_ +0.032 *
Disturbance (=2)	0.420	+0.006 **	+<0.001 ***	0.270	−0.027 *
Rural–urban (=2)	0.607	0.449	−0.003 **	0.927	0.566
Random effect	0.997	0.494	0.487	0.573	0.716
N=	67	277	306	127	93

^(a)^ Flocks of diving ducks with three individuals had a significantly longer FID and those with two individuals had a near-significant longer FID than flocks of one individual (*p* = 0.082).

**Table 3:** *p* values in the GLMM analyses with explanatory factors possibly affecting log_10_FID in waterbird species with a minimum of 50 observations. If significant, the direction of the change is given by + or −. The effects of the explanatory variables are compared with the lowest value (=1) (see Table 1). For flock size, six different categories are compared with one individual and the lowest *p*-value is given. Significant results for the explanatory variables are in bold and * = *p* < 0.05, and *** = *p* < 0.001 for explanatory variables.

Variable	Eurasian Teal	Mallard	Common Eider	Common Goldeneye	Eurasian Oystercatcher
Corrected model	**+0.002**	**+<0.001**	**+<0.001**	**+<0.001**	**+0.004**
Intercept	**<0.001**	**<0.001**	**<0.001**	**<0.001**	**<0.001**
N of researchers (>1)	−<0.001 ***	−<0.001 ***	0.951	0.121	0.708
Open-narrow (=2)	0.464	−<0.001 ***	−0.011 *	0.939	−<0.001 ***
Flock size (2–7)	_Min_0.181	_Min_0.075	0.319	_Min_**+<0.001** ^(a)^***	**_Min_−0.019** ^(b)^*
Disturbance (=2)	0.441	0.169	0.236	+<0.001 ***	0.068
Rural–urban (=2)	0.335	0.380	−<0.001 ***	0.264	0.273
Random effect	0.911	0.597	0.604	0.512	0.710
N=	45	201	163	130	55

^(a)^ Flocks of common goldeneyes with three individuals had a significantly longer FID and those with a flock size category 5 (5–10 individuals) tended to have a longer FID than single individuals (*p* = 0.055). ^(b)^ Eurasian oystercatcher flocks with two individuals had a shorter FID than one individual.

**Table 4:** Results of the MW-U test comparing the FID when birds of different groups flew or walked/swam when fleeing from an approaching human without a dog. + in front of the *p* value means flying birds had the longest FID. Significant results are in bold and * = *p* < 0.05, ** = *p* < 0.01, and *** = *p* < 0.001.

Bird Group	z	*n*	*p*
Swans and geese	−2.064	50	+0.039 *
Dabbling ducks	−5.859	190	+<0.001 ***
Diving ducks	−2.970	100	+0.003 **
Mergansers	−0.215	12	0.830
Waders	−0.393	77	0.695
Gulls	−0.881	71	0.378

**Table 5:** Spearman correlations (R_s_) between the FID and the distance at which the birds stopped normal activity, how far the birds moved from their starting point, and the length of time it took before the birds returned to the starting points when birds were approached by pedestrians on land with or without a leashed dog. No data means that *n* < 10, and * = *p* < 0.05, ** = *p* < 0.01, and *** = *p* < 0.001. Non-significant is written as ns, and for near-significance, the *p* value is given. Significant results are written in bold.

	Stopped Normal Activity	Distance Moved	Time to Return
Swans and geese	0.818, *n* = 17 ***	0.082, *n* = 44, ns	No data
Dabbling ducks	0.974, *n* = 54 ***	0.463, *n* = 117 ***	0.488, *n* = 15, *p* = 0.065
Diving ducks	0.938, *n* = 94 ***	0.269, *n* = 177 ***	−0.181, *n* = 64, ns
Mergansers	Lack of data	0.208, *n* = 19	No data
Waders	0.916, *n* = 29 ***	0.159, *n* = 64, ns	No data
Gulls	No data	−0.084, *n* = 54, ns	No data
Eurasian teal	No data	0.394, *n* = 10, ns	No data
Mallard	0.973, *n* = 52 ***	0.479, *n* = 100 ***	0.488, *n* = 15, *p* = 0.065
Common eider	0.967, *n* = 51 ***	0.243, *n* = 94 *	−0.274, *n* = 49, *p* = 0.056
Common goldeneye	0.898, *n* = 41 ***	195, *n* = 72, ns	−0.082, *n* = 14, ns
Eurasian oystercatcher	0.797, *n* = 11 **	0.033, *n* = 28, ns	No data

## Data Availability

The data presented in this study are available on request from the author.

## References

[1] Sekercioglu C.H. (2006). Increasing awareness of avian ecological function. Trends Ecol. Evol..

[2] Methorst J. (2024). Positive relationship between bird diversity and human mental health: An analysis of repeated cross-sectional data. Lancet Planet. Health.

[3] Furness R.W., Greenwood J.J.D. (1993). Birds as Monitors of Environmental Change.

[4] Gregory R.D., Noble D., Field R., Marchant J., Raven M., Gibbons D.W. (2003). Using birds as indicators of biodiversity. Ornis Hung..

[5] Lees A.C., Haskell L., Allinson T., Bezeng S.B., Burfield I.J., Renjifo L.M., Rosenberg K.V., Viswanathan A., Butchart S.H.M. (2022). State of theWorld’s Birds. Annu. Rev. Environ. Resour..

[6] Brlík V., Šilarová E., Škorpilová J., Alonso H., Anton M., Aunins A., Benkö Z., Biver G., Busch M., Chodkiewicz T. (2021). Long-term and large-scale multispecies dataset tracking population changes of common European breeding birds. Sci. Data.

[7] Sauer J.R., Pardieck K.L., Ziolkowski D.J., Smith A.C., Hudson M.A.R., Rodriguez V., Berlanga H., Niven D.K., Link W.A. (2017). The first 50 years of the North American Breeding Bird Survey. Condor.

[8] Rosenberg K.V., Dokter A.M., Blancher P.J., Sauer J.R., Smith A.C., Smith P.A., Stanton J.C., Panjabi A., Helft L., Parr M. (2019). Decline of the North American avifauna. Science.

[9] Burns F., Eaton M.A., Burfield I.J., Klvariova A., Silarova E., Staneva A., Gregory R.D. (2021). Abundance decline in the avifauna of the European Union reveals cross-continental similarities in biodiversity change. Ecol. Evol..

[10] BirdLife-International (2022). State of the World’s Birds 2022: Insights and Solutions for the Biodiversity Crisis.

[11] IPBES (2019). Global Assessment Report of the Intergovernmental Science-Policy Platform on Biodiversity and Ecosystem Services.

[12] Gurevitch J., Padilla D.K. (2004). Are invasive species a major cause of extinctions?. Trends Ecol. Evol..

[13] Robinson S.K., Thompson F.R., Donovan T.M., Whitehead D.R., Faaborg J. (1995). Regional forest fragmentation and the nesting success of migratory birds. Science.

[14] Husby M., Hoset K., Butler S. (2021). Non-random sampling along rural–urban gradients may reduce reliability of multi-species farmland bird indicators and their trends. Ibis.

[15] Husby M. (2024). Shoreline translocation during road expansion was successful for most waterbirds but not for waders. Land.

[16] Forman R.T.T., Alexander L.E. (1998). Roads and their major ecological effects. Annu. Rev. Ecol. Syst..

[17] Erritzøe J., Mazgajski T.D., Rejt L. (2003). Bird casualties on European roads—A review. Acta Ornithol..

[18] Burns F., Eaton M.A., Barlow K.E., Beckmann B.C., Brereton T., Brooks D.R., Brown P.M.J., Fulaij N.A., Gent T., Henderson I. (2016). Agricultural management and climatic change are the major drivers of biodiversity change in the UK. PLoS ONE.

[19] Kleijn D., Schekkerman H., Dimmers W.J., Van Kats R.J.M., Melman D., Teunissen W.A. (2010). Adverse effects of agricultural intensification and climate change on breeding habitat quality of Black-tailed Godwits Limosa l. limosa in the Netherlands. Ibis.

[20] Butler S.J., Boccaccio L., Gregory R.D., Voříšek P., Norris K. (2010). Quantifying the impact of land-use change to European farmland bird populations. Agric. Ecosyst. Environ..

[21] Stoate C., Baldi A., Beja P., Boatman N.D., Herzon I., van Doorn A., de Snoo G.R., Rakosy L., Ramwell C. (2009). Ecological impacts of early 21st century agricultural change in Europe—A review. J. Environ. Manag..

[22] Reif J., Voříšek P., Stastny K., Bejcek V., Petr J. (2008). Agricultural intensification and farmland birds: New insights from a central European country. Ibis.

[23] Wretenberg J., Lindstrom A., Svensson S., Part T. (2007). Linking agricultural policies to population trends of Swedish farmland birds in different agricultural regions. J. Appl. Ecol..

[24] Fraixedas S., Lindén A., Meller K., Lindström Å., Keišs O., Kålås J.A., Husby M., Leivits A., Leivits M., Lehikoinen A. (2017). Substantial decline of Northern European peatland bird populations: Consequences of drainage. Biol. Conserv..

[25] Wade A.S.I., Barov B., Burfield I.J., Gregory R.D., Norris K., Butler S.J. (2013). Quantifying the detrimental impacts of land-use and management change on European forest bird populations. PLoS ONE.

[26] Newton I. (1998). Population Limitation in Birds.

[27] Ferns P. (1992). Bird Life of Coasts and Estuaries.

[28] Both C., van Turnhout C.A.M., Bijlsma R.G., Siepel H., Van Strien A.J., Foppen R.P.B. (2010). Avian population consequences of climate change are most severe for long-distance migrants in seasonal habitats. Proc. R. Soc. B-Biol. Sci..

[29] Visser M.E., Gienapp P., Husby A., Morrisey M., de la Hera I., Pulido F., Both C. (2015). Effects of spring temperatures on the strength of selection on timing of reproduction in a long-distance migratory bird. PLoS Biol..

[30] Virkkala R., Lehikoinen A. (2017). Birds on the move in the face of climate change: High species turnover in northern Europe. Ecol. Evol..

[31] Stephens P.A., Mason L.R., Green R.E., Gregory R.D., Sauer J.R., Alison J., Aunins A., Brotons L., Butchart S.H.M., Campedelli T. (2016). Consistent response of bird populations to climate change on two continents. Science.

[32] Pearce-Higgins J.W., Green R.E. (2014). Birds and Climate Change: Impacts and Conservation Responses.

[33] Dunn P.O., Møller A.P. (2019). Effects of Climate Change on Birds.

[34] Watson R.T., Kolar P.S., Ferrer M., Nygard T., Johnston N., Hunt W.G., Smit-Robinson H.A., Farmer C.J., Huso M., Katzner T.E. (2018). Raptor interactions with wind energy: Case studies from around the world. J. Raptor Res..

[35] Estellés-Domingo I., López-López P. (2024). Effects of wind farms on raptors: A systematic review of the current knowledge and the potential solutions to mitigate negative impacts. Anim. Conserv..

[36] Peschko V., Mendel B., Muller S., Markones N., Mercker M., Garthe S. (2020). Effects of offshore windfarms on seabird abundance: Strong effects in spring and in the breeding season. Mar. Environ. Res..

[37] Perold V., Ralston-Paton S., Ryan P. (2020). On a collision course? The large diversity of birds killed by wind turbines in South Africa. Ostrich.

[38] Husby M., Pearson M. (2022). Wind farms and power lines have negative effects on territory occupancy in Eurasian eagle owls (*Bubo bubo*). Animals.

[39] Husby M. (2024). Wind farms and power lines reduced the territory status and probability of fledgling production in the Eurasian goshawk Accipiter gentilis. Diversity.

[40] Tolvanen A., Routavaara H., Jokikokko M., Rana P. (2023). How far are birds, bats, and terrestrial mammals displaced from onshore wind power development?—A systematic review. Biol. Conserv..

[41] Nebel C., Stjernberg T., Tikkanen H., Laaksonen T. (2024). Reduced survival in a soaring bird breeding in wind turbine proximity along the northern Baltic Sea coast. Biol. Conserv..

[42] Marques A.T., Batalha H., Bernardino J. (2021). Bird displacement by wind turbines: Assessing current knowledge and recommendations for future studies. Birds.

[43] Grilo C., Koroleva E., Andrasik R., Bil M., Gonzalez-Suarez M. (2020). Roadkill risk and population vulnerability in European birds and mammals. Front. Ecol. Environ..

[44] Husby M. (2016). Factors affecting road mortality in birds. Ornis Fenn..

[45] Brockie R.E., Sadleir R.M.F.S., Linklater W.L. (2009). Long-term wildlife road-kill counts in New Zealand. N. Z. J. Zool..

[46] Forman R.T.T. (2000). Estimate of the area affected ecologically by the road system in the United States. Conserv. Biol..

[47] Reijnen R., Foppen R., Terbraak C., Thissen J. (1995). The effects of car traffic on breeding bird populations in woodland. 3. Reduction of density in relation to the proximity of main roads. J. Appl. Ecol..

[48] Bishop C.A., Brogan J.M. (2013). Estimates of Avian Mortality Attributed to Vehicle Collisions in Canada. Avian Conserv. Ecol..

[49] Loss S.R., Will T., Marra P.P. (2014). Estimation of bird-vehicle collision mortality on US roads. J. Wildl. Manag..

[50] Burger J. (1986). The effect of human activity on shorebirds in two coastal bays in northeastern United States. Environ. Conserv..

[51] Burger J. (1981). The effect of human activity on birds at a coastal bay. Biol. Conserv..

[52] Blumstein D.T., Gil D., Brumm H. (2013). Attention, habituation, and antipredator behaviour: Implications for urban birds. Avian Urban Ecology.

[53] Blumstein D.T., Anthony L.L., Harcourt R., Ross G. (2003). Testing a key assumption of wildlife buffer zones: Is flight initiation distance a species-specific trait?. Biol. Conserv..

[54] Møller A.P., Liang W., Samia D.S.M. (2019). Flight initiation distance, color and camouflage. Curr. Zool..

[55] Diaz M., Møller A.P., Flensted-Jensen E., Grim T., Ibanez-Alamo J.D., Jokimaki J., Marko G., Tryjanowski P. (2013). The geography of fear: A latitudinal gradient in anti-predator escape distances of birds across Europe. PLoS ONE.

[56] Møller A.P. (2008). Flight distance and blood parasites in birds. Behav. Ecol..

[57] Morelli F., Benedetti Y., Díaz M., Grim T., Ibáñez-Alamo J.D., Jokimäki J., Kaisanlahti-Jokimäki M.L., Tätte K., Markó G., Jiang Y.T. (2019). Contagious fear: Escape behavior increases with flock size in European gregarious birds. Ecol. Evol..

[58] Morelli F., Mikula P., Blumstein D.T., Diaz M., Marko G., Jokimaki J., Kaisanlahti-Jokimaeki M.L., Floigl K., Abou Zeid F., Siretckaia A. (2022). Flight initiation distance and refuge in urban birds. Sci. Total Environ..

[59] Møller A.P., Tryjanowski P. (2014). Direction of approach by predators and flight initiation distance of urban and rural populations of birds. Behav. Ecol..

[60] Swarthout E.C.H., Steidl R.J. (2001). Flush responses of Mexican spotted owls to recreationists. J. Wildl. Manag..

[61] Price M.L. (2008). The impact of human disturbance on birds: A selective review. Aust. Zool..

[62] Burger J. (1991). Foraging behavior and the effect of human disturbance on the piping plover (*Charadrius melodus*). J. Coast. Res..

[63] Burger J., Carlucci S.A., Jeitner C.W., Niles L. (2007). Habitat choice, disturbance, and management of foraging shorebirds and gulls at a migratory stopover. J. Coast. Res..

[64] Palacios E., Vargas J., Fernández G., Reiter M.E. (2022). Impact of human disturbance on the abundance of non-breeding shorebirds in a subtropical wetland. Biotropica.

[65] Burger J., Gochfeld M. (1981). Discrimination of the threat of direct versus tangential approach to the nest by incubating herring and great black-backed gulls. J. Comp. Physiol. Psychol..

[66] Burger J., Gochfeld M. (1990). Risk discrimination of direct versus tangential approach by basking black iguanas (Ctenosaura similis): Variation as a function of human exposure. J. Comp. Psychol..

[67] Blumstein D.T. (2003). Flight-initiation distance in birds is dependent on intruder starting distance. J. Wildl. Manag..

[68] Mayer M., Natusch D., Frank S. (2019). Water body type and group size affect the flight initiation distance of European waterbirds. PLoS ONE.

[69] Samia D.S.M., Nomura F., Blumstein D.T. (2013). Do animals generally flush early and avoid the rush? A meta-analysis. Biol. Lett..

[70] Møller A.P. (2015). The value of a mouthful: Flight initiation distance as an opportunity cost. Eur. J. Ecol..

[71] de Resende N.C., Teixeira C.P., de Azevedo C.S. (2024). Flight initiation distance in an urban bird: Influence of the number of people, gaze orientation, and bird behavior. Birds.

[72] Radkovic A.Z., Van Dongen W.F.D., Kirao L., Guay P.J., Weston M.A. (2019). Birdwatchers evoke longer escape distances than pedestrians in some African birds. J. Ecotourism.

[73] Butler P.J. (1991). Exercise in birds. J. Exp. Biol..

[74] Dormann C.F., Elith J., Bacher S., Buchmann C., Carl G., Carre G., Marquez J.R.G., Gruber B., Lafourcade B., Leitao P.J. (2013). Collinearity: A review of methods to deal with it and a simulation study evaluating their performance. Ecography.

[75] IBM (2021). IBM SPSS Statistics.

[76] Lo S., Andrews S. (2015). To transform or not to transform: Using generalized linear mixed models to analyse reaction time data. Front. Psychol..

[77] Bolker B.M., Brooks M.E., Clark C.J., Geange S.W., Poulsen J.R., Stevens M.H.H., White J.S.S. (2009). Generalized linear mixed models: A practical guide for ecology and evolution. Trends Ecol. Evol..

[78] Burnham K.P., Anderson D.R. (2002). Model Selection and Multimodel Inference. A Practical Information-Theoretic Approach.

[79] Zuur A.F., Ieno E.N., Elphick C.S. (2010). A protocol for data exploration to avoid common statistical problems. Methods Ecol. Evol..

[80] Mundry R. (2011). Issues in information theory-based statistical inference-a commentary from a frequentist’s perspective. Behav. Ecol. Sociobiol..

[81] Blumstein D.T. (2006). Developing an evolutionary ecology of fear: How life history and natural history traits affect disturbance tolerance in birds. Anim. Behav..

[82] Collop C., Stillman R.A., Garbutt A., Yates M.G., Rispin E., Yates T. (2016). Variability in the area, energy and time costs of wintering waders responding to disturbance. Ibis.

[83] Chiatante G., Carere C. (2024). Flight initiation distance in waterbirds of two coastal wetlands with different protection regimes. Rend. Lincei.-Sci. Fis. Nat..

[84] Ekanayake K.B., Gnanapragasam J.J., Ranawana K., Vidanapathirana D.R., Abeyawardhana U.T., Fernando C., McQueen A., Weston M.A., Symonds M.R.E. (2022). Ecological and environmental predictors of escape among birds on a large tropical island. Behav. Ecol. Sociobiol..

[85] Reynolds C., Henry D.A.W., Tye D.R.C., Tye N.D. (2021). Defining separation zones for coastal birds at a wetland of global importance. Wildl. Res..

[86] Weston M.A., McLeod E.M., Blumstein D.T., Guay P.J. (2012). A review of flight-initiation distances and their application to managing disturbance to Australian birds. Emu-Austral Ornithol..

[87] Samia D.S.M., Nakagawa S., Nomura F., Rangel T.F., Blumstein D.T. (2015). Increased tolerance to humans among disturbed wildlife. Nat. Commun..

[88] Samia D.S.M., Møller A.P., Blumstein D.T. (2015). Brain size as a driver of avian escape strategy. Sci. Rep..

[89] Madsen J., Cracknell G., Fox T. (1999). Goose Populations of the Western Palearctic. A Review of Status and Distribution.

[90] Kersten M., Piersma T. (1987). High levels of energy expenditure in shorebirds—Metabolic adaptations to an energetically expensive way of life. Ardea.

[91] Burger J., Gochfeld M. (1998). Effects of ecotourists on bird behaviour at Loxahatchee national wildlife refuge, Florida. Environ. Conserv..

[92] Shuai L.Y., Morelli F., Mikula P., Benedetti Y., Weston M.A., Ncube E., Tarakini T., Díaz M., Markó G., Jokimäki J. (2024). A meta-analysis of the relationship between flock size and flight initiation distance in birds. Anim. Behav..

[93] Burger J., Gochfeld M. (1991). Human activity influence and diurnal and nocturnal foraging of sanderlings (*Calidris alba*). Condor.

[94] Elafri A., Halassi I., Boutabia L., Telailia S. (2022). Responses of shorebirds to human disturbance at exposed sandy beaches of north-eastern Algeria. Int. J. Ecol. Dev..

[95] Glover H.K., Weston M.A., Maguire G.S., Miller K.K., Christie B.A. (2011). Towards ecologically meaningful and socially acceptable buffers: Response distances of shorebirds in Victoria, Australia, to human disturbance. Landsc. Urban Plan..

[96] Scarton F. (2018). Flight initiation distances in relation to pedestrian and boat disturbance in five species of waders breeding in a Mediterranean lagoon. Rev. Ecol.-Terre Vie.

[97] Miller S.G., Knight R.L., Miller C.K. (2001). Wildlife responses to pedestrians and dogs. Wildl. Soc. Bull..

[98] Novcic I. (2022). Behavioural responses of grey herons Ardea cinerea and great egrets Ardea alba to human-caused disturbance. J. Vertebr. Biol..

[99] Lafferty K.D. (2001). Disturbance to wintering western snowy plovers. Biol. Conserv..

[100] Møller A.P. (2008). Flight distance of urban birds, predation, and selection for urban life. Behav. Ecol. Sociobiol..

[101] Mikula P. (2014). Pedestrian density influences flight distances of urban birds. Ardea.

[102] Yin L.Q., Wang C., Han W.J., Zhang C. (2023). Birds’ flight initiation distance in residential areas of Beijing are lower than in pristine environments: Implications for the conservation of urban bird diversity. Sustainability.

[103] Zhou B., Liang W. (2020). Avian escape responses to observers wearing clothing of different colors: A comparison of urban and rural populations. Glob. Ecol. Conserv..

[104] Samia D.S.M., Blumstein D.T., Díaz M., Grim T., Ibáñez-Alamo J.D., Jokimäki J., Tätte K., Markó G., Tryjanowski P., Møller A.P. (2017). Rural-urban differences in escape behavior of European birds across a latitudinal gradient. Front. Ecol. Evol..

[105] Azaki B.D.A., Cresswell W. (2021). Level of local human disturbance and feeding state determines escape behaviour in Eurasian Oystercatchers. Ethology.

[106] Xu W.Y., Gong Y., Wang H.T. (2021). Alert time reflects the negative impacts of human disturbance on an endangered bird species in Changbai Mountain, China. Glob. Ecol. Conserv..

[107] Nepali A., Katuwal H.B., Kc S., Regmi S., Sharma H.P. (2024). Flight initiation distance and bird tolerance to humans in rural and urban habitats. R. Soc. Open Sci..

[108] Trulio L.A., Sokale J. (2008). Foraging shorebird response to trail use around San Francisco bay. J. Wildl. Manag..

[109] Glover H.K., Guay P.J., Weston M.A. (2015). Up the creek with a paddle; avian flight distances from canoes versus walkers. Wetl. Ecol. Manag..

[110] Mo M., Bonatakis K. (2022). An examination of trends in the growing scientific literature on approaching wildlife with drones. Drone Syst. Appl..

[111] Howell L.G., Allan B.M., Driscoll D.A., Ierodiaconou D., Doran T.A., Weston M.A. (2023). Attenuation of responses of waterbirds to repeat drone surveys involving a sequence of altitudes and drone types: A case study. Drones.

[112] Francis R.J., Lyons M.B., Kingsford R.T., Brandis K.J. (2020). Counting Mixed Breeding Aggregations of Animal Species Using Drones: Lessons from Waterbirds on Semi-Automation. Remote Sens..

[113] Corregidor-Castro A., Scarton F., Panzarin L., Verza E., Valle R.G. (2022). Faster and better: Comparison between traditional and drone monitoring in a cryptic species, the Purple Heron *Ardea purpurea*. Acta Ornithol..

[114] Jarrett D., Calladine J., Cotton A., Wilson M.W., Humphreys E. (2020). Behavioural responses of non-breeding waterbirds to drone approach are associated with flock size and habitat. Bird Stud..

[115] Møller A.P. (2008). Flight distance and population trends in European breeding birds. Behav. Ecol..

[116] Lilleyman A., Franklin D.C., Szabo J.K., Lawes M.J. (2016). Behavioural responses of migratory shorebirds to disturbance at a high-tide roost. Emu-Austral Ornithol..

[117] Goss-Custard J.D., Jenyon R.A., Jones R.E., Newbery P.E., Williams R.L.B. (1977). Ecology of the Wash 2. Seasonal variation in feeding conditions of wading birds (*Charadrii*). J. Appl. Ecol..

[118] Urfi A.J., Goss-Custard J.D., Durell S. (1996). The ability of oystercatchers Haematopus ostralegus to compensate for lost feeding time: Field studies on individually marked birds. J. Appl. Ecol..

[119] Tätte K., Møller A.P., Mänd R. (2018). Towards an integrated view of escape decisions in birds: Relation between flight initiation distance and distance fled. Anim. Behav..

[120] Husby M. (1986). On the adaptive value of brood reduction in birds: Experiments with the magpie Pica pica. J. Anim. Ecol..

[121] Colwell M.A. (2010). Shorebird Ecology, Conservation, and Management.

[122] Livezey K.B., Fernandez-Juricic E., Blumstein D.T. (2016). Database of bird flight initiation distances to assist in estimating effects from human disturbance and delineating buffer areas. J. Fish Wildl. Manag..

[123] Mikula P., Tomásek O., Romportl D., Aikins T.K., Avendano J.E., Braimoh-Azaki B.D.A., Chaskda A., Cresswell W., Cunningham S.J., Dale S. (2023). Bird tolerance to humans in open tropical ecosystems. Nat. Commun..

[124] Jiang Y., Møller A.P. (2017). Escape from predators and genetic variance in birds. J. Evol. Biol..

[125] Møller A.P., Erritzøe J. (2010). Flight distance and eye size in birds. Ethology.

[126] Meager J.J., Schlacher T.A., Nielsen T. (2012). Humans alter habitat selection of birds on ocean-exposed sandy beaches. Divers. Distrib..

[127] Lafferty K.D., Goodman D., Sandoval C.P. (2006). Restoration of breeding by snowy plovers following protection from disturbance. Biodivers. Conserv..

[128] Ma A.T.H., Ng S.L., Cheung L.T.O., Lam T.W.L. (2022). The effectiveness of bird hides in mitigating recreational disturbances of birdwatchers. J. Nat. Conserv..

[129] Clausen K.K., Bregnballe T. (2022). Mapping important roost sites for waders to alleviate human-waterbird conflicts in the Danish Wadden Sea. Ocean. Coast. Manag..

[130] Mahar N., Dobriyal P., Badola R., Hussain S.A. (2024). Tourism on the roof of the world: Socio-ecological impacts of tourism on the Indian Trans-Himalaya. Land Use Pol..

